# Efficacy of a new surgical indicator for lung cancer with interstitial lung disease based on the gender–age–physiology staging system

**DOI:** 10.1007/s10147-026-03164-2

**Published:** 2026-08-21

**Authors:** Yoshito Imamura, Keita Nakanishi, Heng Huang, Minoru Sugihara, Taiki Ryo, Yasuhisa Ichikawa, Tatsuya Masuda, Hirofumi Takenaka, Yuta Kawasumi, Hiroki Watanabe, Yuka Kadomatsu, Harushi Ueno, Taketo Kato, Shota Nakamura, Tetsuya Mizuno, Toyofumi Fengshi Chen-Yoshikawa

**Affiliations:** Department of Thoracic Surgery, Nagoya University Graduate School of Medicine, 65 Tsurumai-cho, Showa-ku, Nagoya, 466-8550 Japan

**Keywords:** Interstitial lung disease, Lung cancer, ILD-gender–age–physiology stage, Prognosis

## Abstract

**Background:**

Selecting the optimal surgical strategy for lung cancer patients with interstitial lung disease (ILD) remains difficult. This study evaluated the prognostic value of a newly proposed surgical indicator—the predicted ILD Gender–Age–Physiology (GAP) stage—which incorporates predicted postoperative pulmonary function into the conventional ILD-GAP stage.

**Methods:**

This study retrospectively analyzed 125 patients with clinical stage 1 lung cancer and ILD who underwent surgery during 2011–2023. Postoperative outcomes were compared according to surgical procedure and predicted ILD-GAP stage.

**Results:**

The 5-year overall survival (OS) rate was markedly superior in patients who underwent anatomical resection with a predicted ILD-GAP stage ≤ 2 than in those who underwent wedge resection (75.0% vs. 21.3%, *p* < 0.001). In contrast, survival did not differ between anatomical resection with a predicted ILD-GAP stage 3 and wedge resection (27.8% vs. 21.3%, *p* = 0.934). Multivariate analysis identified diffusing capacity of carbon monoxide (*p* = 0.043), anatomical resection with a predicted ILD-GAP stage ≤ 2 (*p* = 0.009), and pathological stage 1 (*p* = 0.035) as independent predictors of prognosis. Furthermore, the predicted ILD-GAP stage provided superior prognostic accuracy for 5-year OS compared with the original ILD-GAP stage (area under the curve: 0.735 vs. 0.653; *p* = 0.040).

**Conclusions:**

The proposed surgical indicator enhanced prognostic stratification in lung cancer patients with ILD. In particular, anatomical lung resection in patients with a predicted ILD-GAP stage ≤ 2 was identified as an independent favorable prognostic factor for 5-year OS, highlighting the importance of surgical planning.

## Introduction

Interstitial lung disease (ILD) has been recognized as a risk factor for lung cancer, with around 6–17% of ILD patients developing lung cancer [1]. However, the management of lung cancer patients with ILD is particularly difficult owing to the fatal risk of acute exacerbation (AE). In fact, one study showed that nearly 10% of lung cancer patients with ILD experienced a postoperative AE within 30 days after surgery, with mortality rates reaching up to 40% [2], Consequently, these patients should be treated following a comprehensive assessment of tumor stage, ILD subtype, and pulmonary function. Recently, several studies have applied the Gender–Age–Physiology (GAP) staging system to evaluate the postoperative prognosis of lung cancer patients with ILD [3, 4]. This staging system is a well-established prognostic indicator for ILD. However, as this method does not evaluate loss of pulmonary function due to lung resection, the optimal surgical strategy for minimizing cancer- and ILD-related mortality in these patients has yet to be unknown. Therefore, we developed a modified prognostic model, namely the predicted ILD-GAP stage, considering loss of pulmonary function at surgery. The primary objective of this study was to investigate the usefulness of this model in lung cancer surgery for patients with ILD.

## Materials and methods

### Ethical considerations

All studies involving human participants were conducted under the ethical standards outlined by institutional committees and the 1964 Declaration of Helsinki, along with its subsequent amendments or equivalent ethical standards. This study was approved by the Ethics Committee of Nagoya University School of Medicine (accession No 2018−0258).

### Patients

**Fig. 1:**
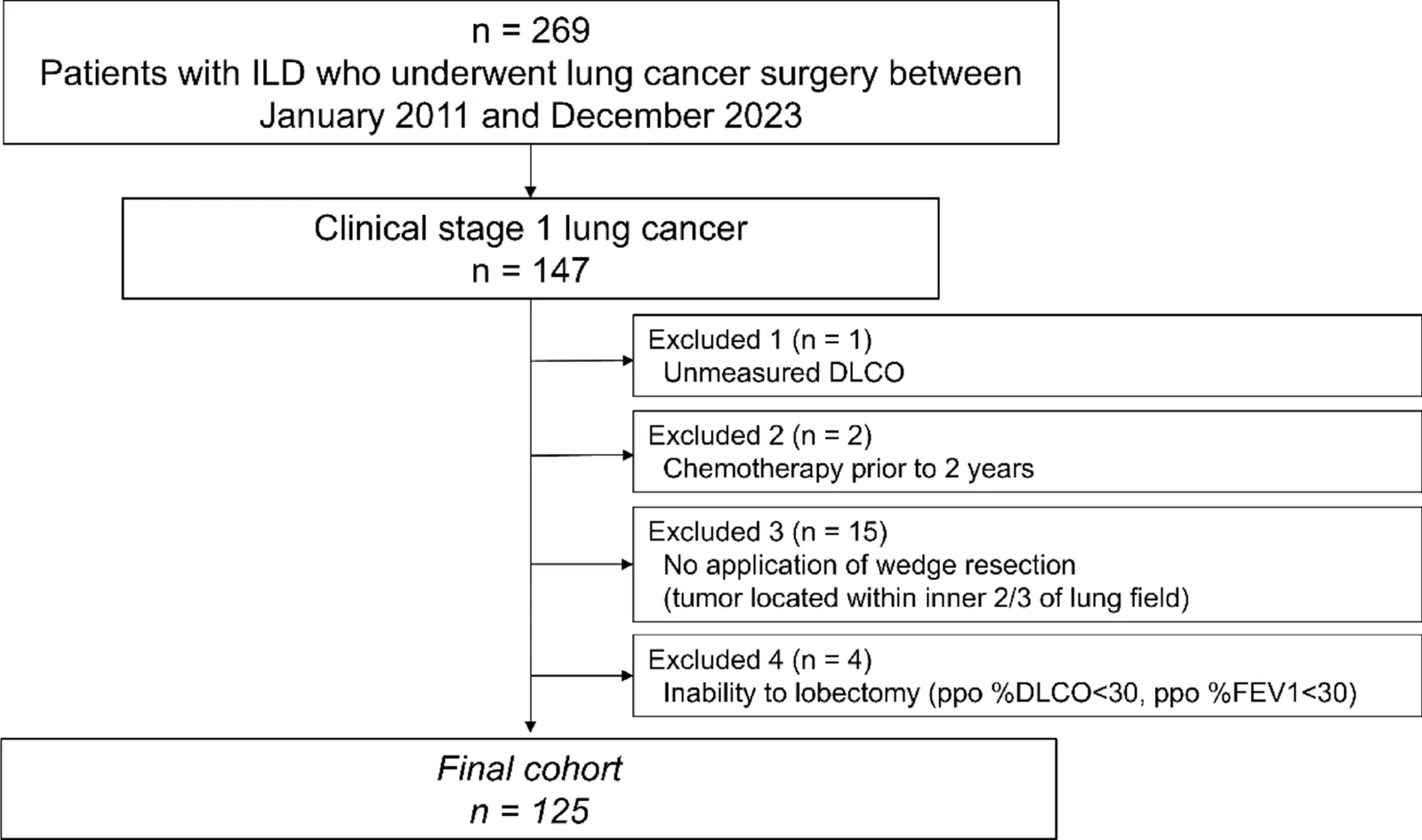
Flow chart showing patient selection. ILD, interstitial lung disease; ppo %DLCO, predicted postoperative diffusing capacity of carbon monoxide; ppo %FEV1, predicted postoperative forced expiratory volume in 1 s

A total of 269 patients with ILD who underwent lung cancer surgery at our hospital from 2011 to 2023 were evaluated. Among them, 147 patients had clinical stage 1 lung cancer. As illustrated in Fig. 1, the following patients were excluded: (1) those with unmeasured diffusing capacity of carbon monoxide (DLCO) (*n* = 1), (2) those who received chemotherapy 2 years prior to study participation (*n* = 2), (3) those in whom wedge resection could not be applied due to the location of the tumor within the inner two-thirds of the lung field (*n* = 15), and (4) those who were ineligible for lobectomy due to a predicted postoperative %DLCO of < 30 or predicted postoperative forced expiratory volume in one second (FEV1) of < 30 (*n* = 4). Ultimately, a total of 125 patients were included in this retrospective analysis.

### Data collection

The following clinical and pathological characteristics were retrieved from the hospital information system and the institutional database: age (at the time of surgery), sex, body mass index, Brinkman index, history of antifibrotic medication, Eastern Cooperative Oncology Group performance status (ECOG PS), medical history, preoperative pulmonary function values, surgical procedure, computed tomography (CT) and positron emission tomography (PET)/CT findings, clinical stage, and pathological diagnosis. The clinicopathologic stage in all patients was assigned following the 9th edition of the tumor, node, and metastasis staging system [5]. Patients with no lymph node enlargement > 1 cm in short diameter or those with a standardized uptake value max < 2.5 on PET/CT were considered cN0 [6, 7]. This study included patients eligible for either anatomical lung resection or wedge resection, with the choice of surgical procedure being determined through a conference led by the thoracic surgeon.

Data on clinical outcomes, recurrence, AE of ILD, antifibrotic drug introduction, postrecurrence treatment, and cause of death were collected and analyzed. Recurrence was diagnosed based on diagnostic imaging, with or without histopathological confirmation. Postoperative recurrence-free survival (RFS) was defined as the interval from the date of surgery to the date of recurrence or last follow-up. Postoperative overall survival (OS) length was defined as the interval from the date of surgery to death from any cause or last follow-up. ILD-AE was defined as an acute, clinically significant respiratory deterioration accompanied by new radiologic abnormalities (i.e., diffuse ground-glass opacities on high-resolution CT) in the absence of other causes, such as heart failure, pulmonary embolism, or aspiration pneumonia [8]. Observations were censored at the last follow-up when the patient was alive or lost to follow-up. The cutoff for data collected was the end of June 2025.

### ILD diagnosis

ILD was divided into IPF, non-IPF, or unclassifiable based on preoperative CT findings. Pursuant to the classification system proposed by the American Thoracic Society (ATS), European Respiratory Society (ERS), Japanese Respiratory Society (JRS), and Latin American Thoracic Association (ALAT) [9], a radiological diagnosis of ILD on high-resolution CT was defined based on one of the following patterns: (1) usual interstitial pneumonia (UIP) pattern, characterized by subpleural and basal predominance, reticular abnormalities, and honeycombing; (2) probable UIP pattern showing subpleural and basal predominance with reticular abnormalities but without definitive honeycombing; and (3) pattern inconsistent with UIP, including features such as upper- or middle-lung predominance, peribronchovascular distribution, extensive ground-glass opacities, profuse micronodules (predominantly in both upper lobes), discrete cysts (multiple, bilateral, and distant from areas of honeycombing), diffuse mosaic attenuation or air trapping involving three or more lobes bilaterally, and lobar or segmental consolidation. IPF was diagnosed based on the official ATS/ERS/JRS/ALAT diagnostic criteria [10] through multidisciplinary discussion (MDD) involving pulmonologists, thoracic radiologists, and pulmonary pathologists. Similarly, unclassifiable ILD and non-IPF ILD were diagnosed through the same MDD process.

### Calculation of the predicted ILD-GAP stage

**Fig. 2:**
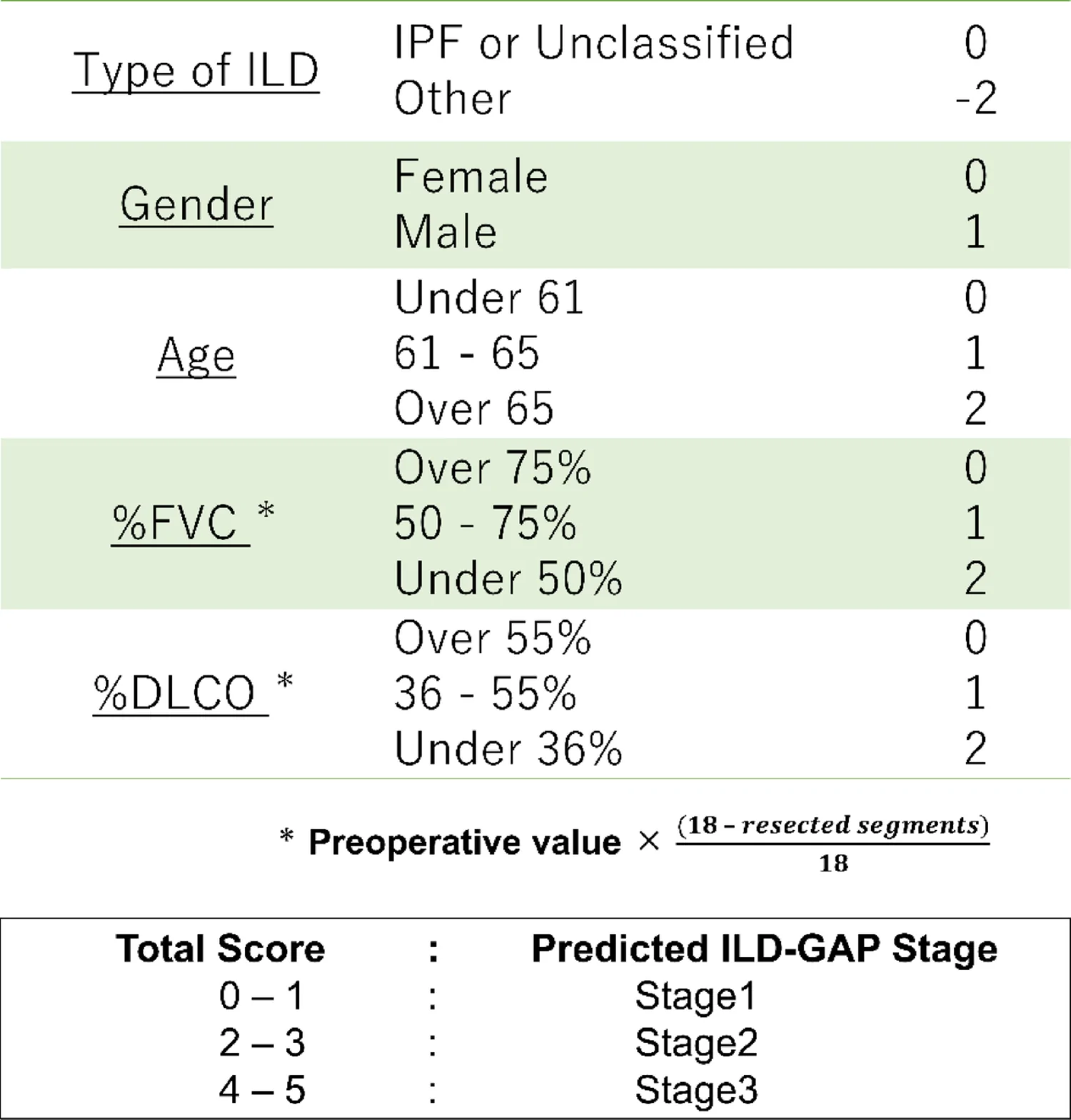
Methods of measuring predicted ILD-GAP stage. ILD, interstitial lung disease; IPF, Idiopathic pulmonary fibrosis; FVC, Forced vital capacity; DLCO, Diffusing capacity of carbon monoxide; GAP, Gender–age–physiology

The predicted ILD-GAP stage was calculated using the same method as the original ILD-GAP stage [11], except that the parameters related to pulmonary function were evaluated using predicted postoperative values. Consequently, the novelty of this study lies in the development of a new surgical indicator for prognostic stratification based on residual postoperative lung function. Considering that the predicted ILD-GAP stage requires the calculation of predicted postoperative pulmonary function, it was applied to patients who had undergone anatomic resection. As illustrated in Fig. 2, the predicted ILD-GAP stage is determined using five factors: ILD subtype (IPF and unclassifiable ILD, 0; non-IPF, − 2), sex (female, 0; male, 1), age (years; ≤60, 0; 61–65, 1; >65, 2), predicted postoperative %forced vital capacity (%FVC) (> 75%, 0; 50–75%, 1; <50%, 2), and predicted postoperative %DLCO (> 55%, 0; 36–55%, 1; ≤35%, 2). Patients with a total score of 0–1, 2–3, and 4–5 points were classified as stage 1, stage 2, and stage 3, respectively. The predicted postoperative pulmonary function was estimated by multiplying the preoperative values by the ratio of the total remaining lungs after surgery (based on a total of 18 bronchopulmonary segments). If the patient had a previous anatomical lung resection, the number of areas was subtracted from 18, while if the patient had a previous wedge resection, the number of areas was calculated as 18 [12]. 

### Statistical analysis

Categorical variables were compared using Fisher’s exact tests. Continuous variables were presented as medians (range) and compared using the Mann–Whitney test. The Kaplan–Meier method was used to estimate survival curves, and the log-rank test was utilized to compare differences in postoperative OS. Univariate and multivariate Cox proportional hazards models were created to estimate hazard ratios (HRs) and 95% confidence intervals (CIs) to identify independent prognostic risk factors. Variables with a *p* value of < 0.05 during univariate analysis were included in the multivariate model. Multicollinearity was assessed by computing the variance inflation factor (VIF) for each predictor. Notably, all variables had a VIF value of < 5, which was considered acceptable. The incidence of ILD exacerbation during the observation period was analyzed using the Fine–Gray hazard model, which accounts for the competing risk of death. The model validity of the original ILD-GAP stage and predicted ILD-GAP stage was assessed by comparing the area under the receiver operating characteristic curve (AUC) for 5-year OS rates, as estimated using the Kaplan–Meier method. All statistical analyses were performed using EZR version 1.68 [13], with *p* values of < 0.05 indicating statistical significance.

## Results

### Patient characteristics and clinical outcomes

The clinicopathological characteristics for the entire cohort are presented in Table 1. A comparison of the wedge resection (*n* = 27), segmentectomy (*n* = 14), and lobectomy groups (*n* = 84) revealed that ECOG PS and %DLCO were significantly worse in the wedge resection group than in the lobectomy group. The clinical outcomes of our cohort are summarized in Table 2. Notably, the proportion of patients who underwent video-assisted thoracic surgery was significantly higher in the wedge resection group (96.3%) than in the segmentectomy (21.4%, *p* < 0.001) and lobectomy groups (24.7%, *p* < 0.001). Pathological stage 0–1B was most frequent observed clinical stage across all groups, though the lobectomy group comprised a significantly higher proportion of patients with stage 2 disease than did the wedge resection group (*p* = 0.021). During the pre-adjustment analysis, recurrence rates and treatment modalities were similar across surgical types. Regarding postrecurrence survival, both the best supportive care group (*n* = 14) and the treatment group (*n* = 18) displayed poor prognoses, with a median survival time of 7.6 and 11.4 months after recurrence, respectively. ILD-related deaths tended to be more frequent in the lobectomy (22.9%) and segmentectomy groups (33.3%) than in the wedge group (7.7%), although the difference did not reach statistical significance during the whole-cohort comparison.

**Table 1:** Patient characteristics

	Wedge resection (n = 27)	Segmentectomy (n = 14)	Lobectomy (n = 84)	*p* value Wedge vs. Segmentectomy Wedge vs. Lobectomy	
Age, median (range)	74 (65–86)	74 (65–83)	72 (38–84)	0.858	0.077
Sex, n (%)					
Female	3 (11.1)	3 (21.4)	16 (19.0)	0.393	0.557
Male	24 (88.9)	11 (78.6)	68 (81.0)		
Smoking, n (%)					
Never-smoker	2 (7.4)	4 (28.6)	4 (4.8)	0.157	0.632
Current- or ex-smoker	25 (92.6)	10 (71.4)	80 (95.2)		
Brinkman Index, median (range)	880 (0–2320)	673 (0–1350)	870 (0–3600)	0.113	0.635
Type of interstitial lung disease, n (%)					
IPF	18 (66.7)	8 (57.1)	46 (54.8)	0.868	0.501
Unclassified	2 (7.4)	1 (7.1)	6 (7.1)		
Non-IPF	7 (25.9)	5 (35.7)	32 (38.1)		
ECOG PS, n (%)					
0	20 (74.1)	12 (85.7)	77 (91.7)	0.692	**0.039**
1	7 (25.9)	2 (14.3)	7 (8.3)		
Simplified comorbidity score, median (range)	9 (2–14)	8 (1–14)	8 (1–17)	0.351	0.098
Clinical stage, n (%)					
1A	10 (37.0)	5 (35.7)	39 (46.4)	1.00	0.505
1B	17 (63.0)	9 64.3)	45 (53.6)		
%FVC, median (range)	99 (66–152)	109 (79–129)	104 (74–142)	0.178	0.057
%DLCO, median (range)	74 (40–115)	78 (63–138)	86 (40–141)	0.226	**0.017**
FEV1/ FVC, median (range)	78 (47–130)	73 (55–83)	74 (59–98)	0.093	0.053
Tumor location, n (%)					
Upper or middle lobe	10 (37.0)	4 (28.6)	35 (41.7)	0.734	0.822
Lower lobe	17 (63.0)	10 (71.4)	49 (58.3)		
Consolidation to tumor ratio, n (%)					
<0.5	0 (0)	1 (7.1)	2 (2.4)	0.341	1.000
≥0.5	27 (100)	13 (92.9)	82 (97.6)		
SUV max, median (range)	6 (1–20)	6 (1–17)	7 (1–22)	0.475	0.853
Preoperative antifibrotic drugs, n (%)	0 (0)	0 (0)	1 (1.2)	1.000	1.000

IPF, Idiopathic pulmonary fibrosis; ECOG PS, Eastern cooperative oncology group performance status; FVC, Forced vital capacity; DLCO, Diffusing capacity of the lung carbon monoxide; FEV1, 1 s Forced expiratory volume; SUV, Standardized uptake value

Bold indicates that the p-value is 0.05 or less

**Table 2:** Details of clinical outcomes of patients in the cohort

	Wedge resection (n = 27)	Segmentectomy (n = 14)	Lobectomy (n = 84)	*p* value Wedge vs. Segmentectomy Wedge vs. Lobectomy	
Surgical approach, n (%)					
VATS	26 (96.3)	3 (21.4)	21 (24.7)	**< 0.001**	**< 0.001**
RATS	0 (0)	5 (35.7)	14 (16.5)		
Thoracotomy	1 (3.7)	6 (42.9)	49 (58.8)		
Pathological stage, n (%)					
1A	10 (37.0)	5 (35.7)	36 (42.9)	0.546	**0.021**
1B	17 (63.0)	8 (57.1)	34 (40.0)		
≥2	0 (0)	1 (7.1)	14 (17.6)		
Pathological type, n (%)					
Adenocarcinoma	6 (22.2)	8 (57.1)	42 (49.4)	0.088	**0.025**
Squamous cell carcinoma	16 (59.3)	5 (35.7)	34 (40.0)		
Other	5 (18.5)	1 (7.1)	8 (10.6)		
ILD-AE, n (%)	3 (11.1)	1 (7.1)	16 (18.8)	1.000	0.557
Postoperative antifibrotic drugs, n (%)	2 (7.4)	0 (0)	2 (2.4)	0.539	0.248
Recurrence, n (%)	8 (29.6)	1 (7.1)	23 (27.1)	0.131	0.810
Initial treatment for recurrence, n (%)					
Chemotherapy or ICI treatment	3 (37.5)	1 (100)	5 (21.7)	1.000	0.384
Radiation therapy	3 (37.5)	0 (0)	6 (26.1)		
Best supportive care	2 (25.0)	0 (0)	12 (52.2)		
Death, n (%)	13 (48.1)	3 (21.4)	35 (41.7)	0.176	0.656
Lung cancer death	5 (38.5)	1 (33.3)	15 (42.9)	0.645	1.000
ILD-related death	1 (7.7)	1 (33.3)	8 (22.9)	1.000	0.450

VATS, Video-assisted thoracic surgery; RATS, Robot-assisted thoracoscopic surgery; ILD-AE, Interstitial lung disease acute exacerbation; ICI, Immune checkpoint inhibitor

Bold indicates that the p-value is 0.05 or less

### Prognosis and OS-related factors

**Fig. 3:**
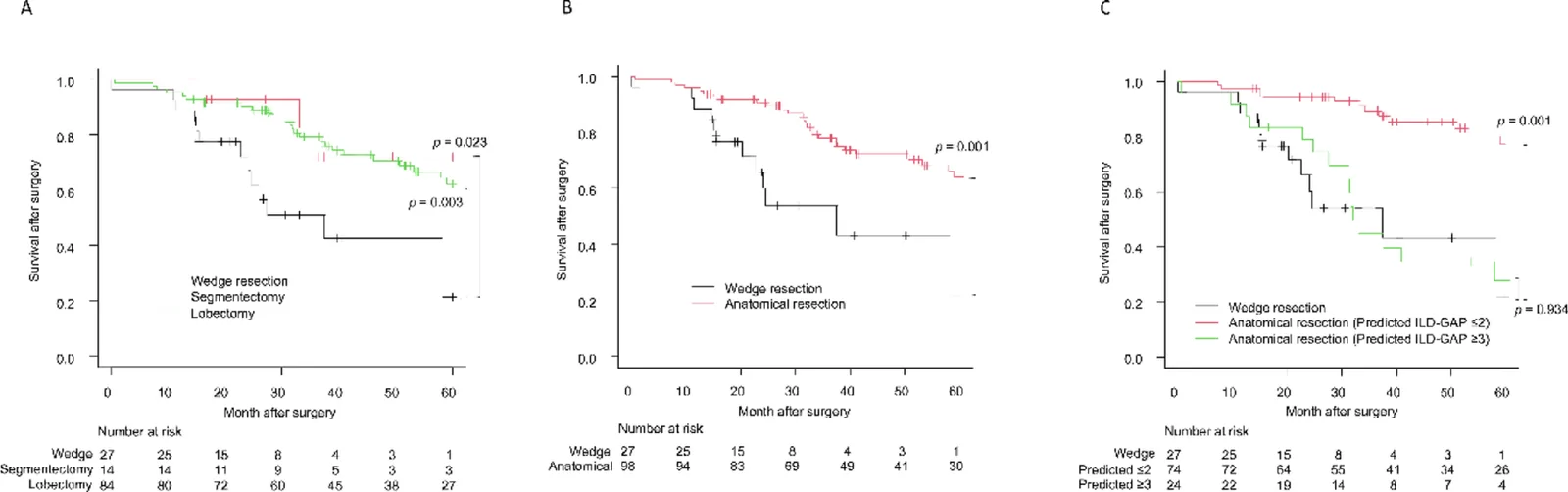
Kaplan–Meier curves showing 5-year overall survival according to surgical type without adjustment for pulmonary function (**a**, **b**) and according to surgical type and predicted ILD-GAP stage (**c**). ILD, Interstitial lung disease; GAP, Gender–age–physiology

The patients included in this study had a median postoperative follow-up duration of 44.9 (interquartile range: 29.4–69.2) months for censored cases. The Kaplan–Meier curves for the 5-year OS rate between the wedge resection, segmentectomy, and lobectomy groups are presented in Fig. 3a. Notably, a significant difference was found between wedge resection and segmentectomy (*p* = 0.023, 5-year OS rate: 21.3% vs. 72.2%; HR: 4.331, 95% CI: 1.221–15.361) or lobectomy (*p* = 0.003, 5-year OS rate: 21.3% vs. 62.2%; HR: 2.730, 95% CI: 1.397–5.333). A comparison of the 5-year OS rate between the wedge resection and anatomical lung resection groups, without adjustment for pulmonary function, revealed significantly poorer prognosis in the wedge resection group (Fig. 3b; *p* = 0.001, 5-year OS rate: 21.3% vs. 62.9%; HR: 2.946, 95% CI: 1.465–5.925). Finally, the Kaplan–Meier curves for 5-year OS based on surgical type and predicted ILD-GAP stage is presented in Fig. 3c. The figure shows that when the severity of ILD is limited to a predicted ILD-GAP stage ≤ 2, patients who underwent anatomical lung resection showed a markedly superior 5-year OS than did those who underwent wedge resection (75.0% vs. 21.3%; *p* < 0.001; HR 0.181, 95% CI 0.080–0.411). In contrast, patients who underwent anatomical lung resection with a predicted ILD-GAP stage 3 showed no significant difference in survival rates compared to those who underwent wedge resection (27.8% vs. 21.3%; *p* = 0.934; HR 0.968, 95% CI 0.449–2.086).

Univariate and multivariate Cox proportional hazards analyses (Table 3) identified three variables independently associated with 5-year OS, including %DLCO (HR = 0.982, 95% CI: 0.964–0.999; *p* = 0.043), anatomical resection in patients with a predicted ILD-GAP of stage ≤ 2 (HR = 0.307, 95% CI: 0.126–0.746; *p* = 0.009), and pathological stage ≥ 2 (HR = 2.709, 95% CI: 1.070–6.858; *p* = 0.035).

**Table 3:** Univariate and multivariate Cox regression analyses for survival after surgery

Variables	n	Univariate			Multivariate		
		Hazard ratio	95% CI	*p* value	Hazard ratio	95% CI	*p* value
Age		1.034	0.994–1.077	0.100			
Sex							
Female	103	1			1		
Male	22	3.024	1.087–8.407	**0.034**	1.730	1.087–8.407	0.382
Type of interstitial lung disease							
IPF	72	1.737	1.223–2.466	**0.002**	1.258	0.847–1.868	0.256
Other	53	1			1		
ECOG PS							
0	109	1					
1	16	1.695	0.818–3.511	0.156			
Simplified comorbidity score		1.108	1.005–1.222	**0.039**	1.057	0.937–1.193	0.367
%FVC		0.972	0.954–0.991	**0.003**	0.985	0.965–1.005	0.147
%DLCO		0.972	0.958–0.986	**< 0.001**	0.982	0.964–0.999	**0.043**
SUV max		1.081	1.021–1.144	**0.008**	1.004	0.933–1.080	0.919
Tumor location							
Upper or middle lobe	49	1					
Lower lobe	76	1.301	0.643–2.641	0.468			
Surgical procedure							
Wedge resection	27	1			1		
Anatomical resection, Predicted ILD-GAP≤2	74	0.250	0.013–0.480	**<0.001**	0.307	0.126–0.746	**0.009**
Anatomical resection, Predicted ILD-GAP≥3	24	1.027	0.531–2.020	0.944	0.591	0.264–1.323	0.203
Surgical approach							
Thoracotomy	56	0.997	0.548–1.814	0.992			
Other	69	1					
pStage							
1A	51	1			1		
1B	59	1.795	0.941–3.425	0.076	1.406	0.662–2.988	0.375
≥2	15	2.333	1.031–5.277	**0.042**	2.709	1.070–6.858	**0.035**
Histology							
Squamous cell carcinoma	55	2.284	1.320–3.930	**0.003**	1.513	0.796–2.875	0.207
Others	70	1			1		

Bold indicates that the p-value is 0.05 or less

### Factors associated with postoperative recurrence and ILD-AE

Table 4 presents the results of the Cox regression analysis for RFS. Among the factors identified as significant in the univariate analysis, IPF (HR = 1.731, 95% CI 1.048–2.859, *p* = 0.032), wedge resection (HR = 3.143, 95% CI: 1.256–7.866, *p* = 0.014), and pathological stage ≥ 2 (HR = 4.705, 95% CI 1.579–14.020, *p* = 0.005) remained as independent predictors of recurrence in the multivariate model.

**Table 4:** Univariate and multivariate Cox regression analyses for recurrence

Variables	n	Univariate			Multivariate		
		Hazard ratio	95% CI	*p* value	Hazard ratio	95% CI	*p* value
Sex							
Female	103	1			1		
Male	22	7.953	1.085–58.290	**0.041**	5.662	0.671–47.750	0.111
Smoking, Brinkman Index		1.001	1.000–1.001	**0.012**	1.000	0.999–1.001	0.894
Type of Interstitial lung disease							
IPF	72	2.021	1.251–3.264	**0.004**	1.731	1.048–2.859	**0.032**
Other	53	1			1		
SUV max		1.067	0.995–1.145	0.070			
Surgical procedure							
Wedge resection	27	2.508	1.093–5.756	**0.030**	3.143	1.256–7.866	**0.014**
Anatomical resection	74	1			1		
pStage							
1A	51	1			1		
1B	59	2.482	0.971–6.344	0.058	1.877	0.715–4.926	0.201
≥2	15	5.426	1.965–14.980	**0.001**	4.705	1.579–14.020	**0.005**
Histology							
Squamous cell carcinoma	55	0.881	0.422–1.841	0.737			
Others	70	1					

Bold indicates that the p-value is 0.05 or less

**Fig. 4:**
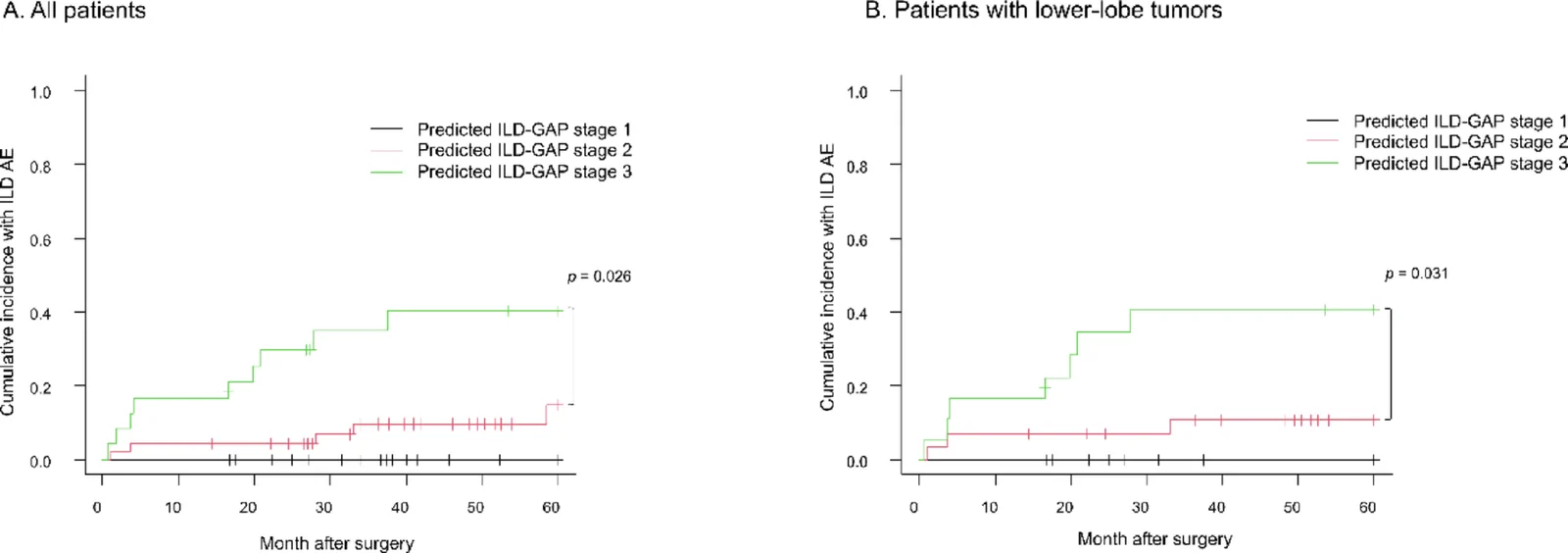
Cumulative incidence of postoperative ILD-AE in the overall anatomical resection group (**a**) and the subgroup with lower-lobe tumor locations (**b**). ILD, Interstitial lung disease; AE, Acute exacerbation; GAP, Gender–age–physiology

Figure 4a illustrates the cumulative incidence of postoperative ILD-AE among patients who underwent anatomical lung resection. Patients with a predicted ILD-GAP stage of 3 exhibited a significantly higher incidence of AE than those with stage 2 (HR = 3.029, 95% CI: 1.142–8.034, *p* = 0.026). Furthermore, a subgroup analysis restricted to patients with lower-lobe tumors revealed a consistent trend, confirming that the predicted ILD-GAP stage remained a significant predictor of AE incidence even within this specific cohort (Stage 3 vs. 2: HR = 4.368, 95% CI: 1.141–16.72, *p* = 0.031; Fig. 4b).

### Comparison of prognostic indicators between the ILD-GAP stage and the predicted ILD-GAP stage

**Fig. 5:**
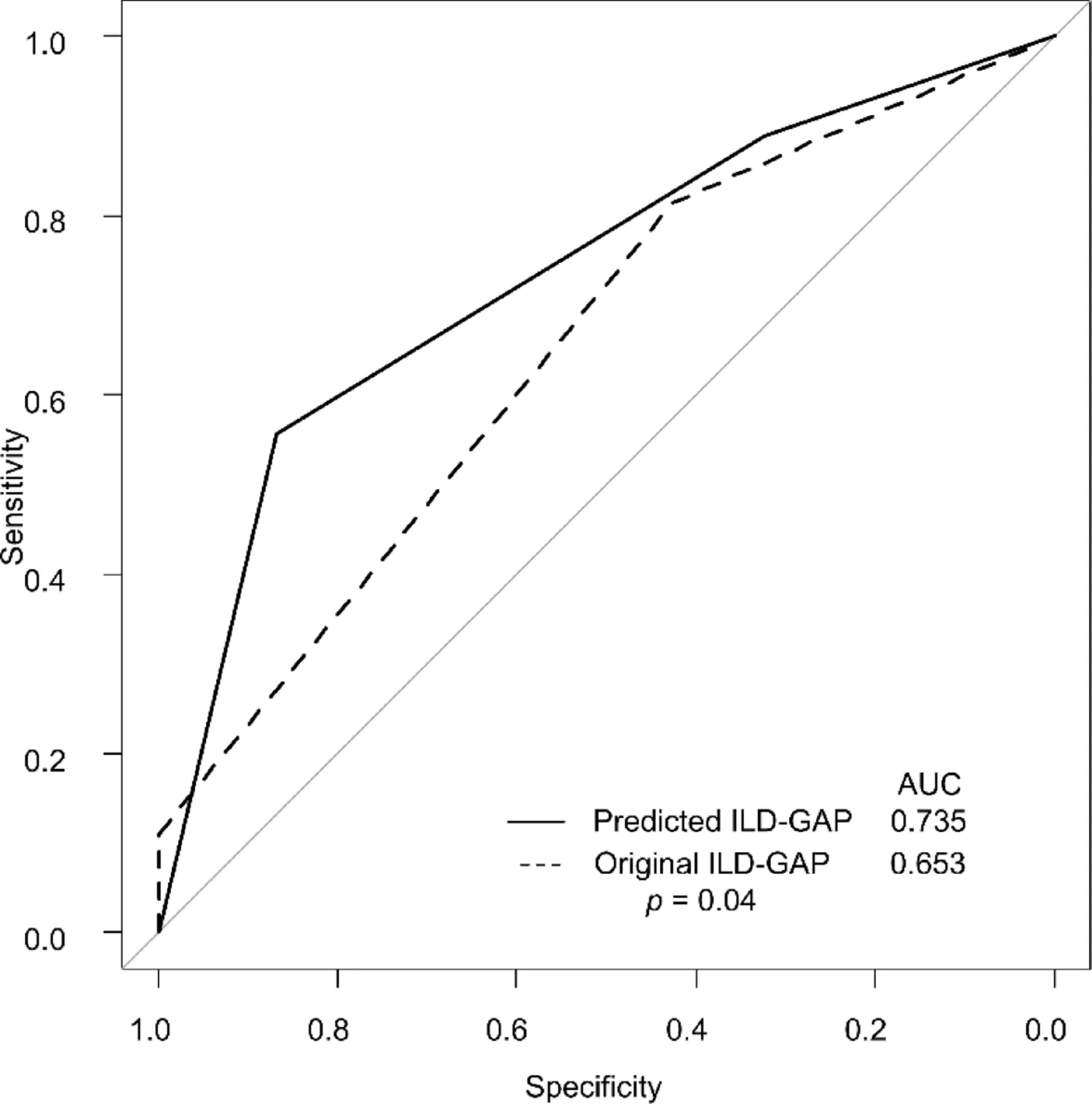
Predictive accuracy of the original ILD-GAP stage and predicted ILD-GAP stage. ILD, Interstitial lung disease; GAP, Gender–age–physiology; AUC, Area under the curve

Table 5 details the differences between the original ILD-GAP stage and the predicted ILD-GAP stage in patients who underwent anatomical lung resection. The predicted ILD-GAP stage is a modified model that includes the predicted postoperative pulmonary function in the original ILD-GAP stage. Among the 58 patients categorized as original ILD-GAP stage 2, 21 patients (36.2%) were reclassified as predicted ILD-GAP stage 3, whereas the remaining 37 patients remained at predicted ILD-GAP stage 2. Patients who were upstaged to predicted ILD-GAP stage 3 had a significantly higher incidence of AE than those who remained at stage 2 (HR = 3.080, 95% CI: 1.120–8.472, *p* = 0.029). Notably, the 5-year OS was significantly lower in those reclassified to predicted ILD-GAP stage 3 than in those who remained at stage 2 (37.7% vs. 81.1%, *p* = 0.030). Figure 5 compares the predictive accuracy of the original ILD-GAP stage and predicted ILD-GAP stage for 5-year OS after anatomical lung resection. The predicted ILD-GAP stage provided significantly better discrimination, achieving an AUC of 0.735 versus 0.653 for the original stage (*p* = 0.040).

**Table 5:** Distribution of predicted ILD-GAP stage and original ILD-GAP stage in cases of anatomical lung resection

		Predicted ILD-GAP stage		
		Stage 1	Stage 2	Stage 3
Original ILD-GAP stage	n	28	46	24
Stage 1	37	28	9	0
Stage 2	58	0	37	21
Stage 3	3	0	0	3

## Discussion

In the current study, we developed the predicted ILD-GAP stage, a novel prognostic model that incorporates predicted postoperative pulmonary function into the original ILD-GAP staging system. Notably, multivariate Cox proportional hazards analysis demonstrated that anatomical lung resection with a predicted ILD-GAP stage ≤ 2 was an independent favorable prognostic factor for 5-year OS. These results indicate that both lung cancer–related and ILD-related deaths significantly impacted the postoperative prognosis of lung cancer patients with ILD.

Among the factors influencing lung cancer–related death, a lung cancer stage of 2 or higher has been reported to be a significantly poorer prognostic factor for OS than stage 1, which is consistent with our findings [4, 14]. Immune checkpoint inhibitors or molecular-targeted therapies are not recommended for lung cancer complicated by ILD due to the risk of AE, while chemotherapeutic regimens are generally limited and some patients are unable to receive treatment due to the deterioration of their condition [15–17]. These findings indicate that careful assessment of pulmonary function and performance of radical resection whenever feasible to minimize the risk of recurrence are imperative in the surgical treatment of lung cancer complicated by ILD.

Regarding ILD severity, the current study demonstrated that the predicted ILD-GAP stage had a higher prognostic accuracy than did the original ILD-GAP stage [14, 18]. This outcome may be explained by the fact that the original ILD-GAP stage is calculated based on preoperative pulmonary function; whereas the predicted ILD-GAP stage is calculated using predicted postoperative pulmonary function. Evaluating the loss of pulmonary function following lung resection can result in the reclassification of some cases to a higher stage than the original ILD-GAP stage. Notably, we observed that patients who were upstaged had a significantly poorer prognosis and a higher incidence of ILD-AE than those who remained at the same stage. This finding indicates that our model provides more detailed and clinically meaningful risk stratification than the original ILD-GAP stage. Therefore, the predicted ILD-GAP stage may be reasonably used for stage 1 lung cancer patients with ILD given that it allows for better prognostic stratification.

Regarding the surgical procedure of early-stage non-small cell lung cancer, studies have shown favorable outcomes with anatomical resection and have recommended this strategy for patients with adequately preserved pulmonary function [19–22]. For lung cancer patients with ILD, wedge resection, a conservative approach that preserves pulmonary function has been reported to be associated with lower the risk of postoperative AE than with anatomical resection [1, 2]. However, no reports have demonstrated a prognostic benefit of limited resection in lung cancer patients with ILD, likely due to the increased risk of tumor recurrence, which offsets the advantages of preserving pulmonary function [1, 23, 24]. Indeed, studies have shown that wedge resection significantly increases the risk of postoperative recurrence in solid-predominant lung cancers (consolidation tumor ratio > 0.5) [25]. Given that the proportion of solid-predominant lung cancers is relatively high in patients with ILD, anatomical resection is considered preferable when pulmonary function is adequate. However, evidence regarding the degree of pulmonary function that can be safely tolerated for anatomical resection remains limited [23]. The current study is the first to demonstrate superior prognosis associated with anatomical resection, when a predicted ILD-GAP stage ≤ 2 is used as the criterion for acceptable pulmonary function.

This study has several limitations worth noting. First, our cohort included only patients with clinical stage 1 lung cancer; hence, the overall sample size was relatively small compared to typical large-scale studies. However, our sample size was comparable to those of previous studies focusing on stage 1 lung cancer with ILD [26] Moreover, our median follow-up of 44.9 months was comparable to that reported in previous same studies (41.0–54.3 months) [4, 14, 18]. Furthermore, fulfillment of the proportional hazards assumption ensures that our survival analysis was not biased by early events. Thus, we were able to conduct a more comprehensive analysis focused on stage 1 lung cancer patients with ILD, which was not feasible in earlier studies. Second, the relatively long study period (2011–2023) may have influenced the results considering that surgical strategies for lung cancer with ILD likely varied over time. Therefore, multivariate analyses were conducted to minimize such bias as much as possible. Third, only a small number of patients included herein received antifibrotic therapy, and the impact of such treatment on prognosis could not be fully evaluated. However, current evidence suggests that the therapeutic benefits of antifibrotic agents remains limited, which warrants further investigation [15]. Another limitation is that lung function in patients with ILD is often heterogeneous; therefore, lung function predicted by our model may not fully correspond to actual postoperative values in certain cases, potentially affecting the validity of the proposed scoring system. However, additional subgroup analysis restricted to patients with lower-lobe tumors—who are most susceptible to lobar functional heterogeneity—revealed that the predicted ILD-GAP stage remained consistently and significantly associated with cumulative ILD-AE incidence, supporting the clinical robustness of the proposed index regardless of tumor location. Finally, regarding the baseline differences between the surgical approach groups, residual confounding may have remained despite multivariable adjustment. Because of the limited sample size, more robust methods such as propensity score matching or inverse probability weighting could not be applied. Further prospective multicenter studies are warranted to address this issue [12]. 

In conclusion, this study is the first to demonstrate that anatomical lung resection in patients with a predicted ILD-GAP stage ≤ 2 represents an independent favorable prognostic factor for 5-year OS. By integrating ILD severity with anticipatory postoperative pulmonary function, the predicted ILD-GAP stage may serve as a valuable prognostic indicator for patients undergoing anatomical lung resection.

## Data Availability

The data used in this study contain personal patient information and are therefore not publicly available. Access to the data may be considered upon reasonable request to the corresponding author and with appropriate ethical approval.
